# Estimated Change in Prevalence and Trends of Childhood Blood Pressure Levels in the United States After Application of the 2017 AAP Guideline

**DOI:** 10.5888/pcd16.180528

**Published:** 2019-01-31

**Authors:** Gulam Muhammed Al Kibria, Krystal Swasey, Atia Sharmeen, Brendan Day

**Affiliations:** 1 Department of Epidemiology and Public Health, School of Medicine, University of Maryland, Baltimore, Baltimore, Maryland; 2 Department of International Health, Bloomberg School of Public Health, Johns Hopkins University, Baltimore, Maryland; 3 School of Community Health and Policy, Morgan State University, Baltimore, Maryland

## Abstract

**Introduction:**

Childhood hypertension is associated with higher risks of cardiovascular disease during adulthood. This study estimated the prevalence of hypertension and high blood pressure among children aged 8 to 17 years in the United States per the 2017 American Academy of Pediatrics (AAP) guideline and compared that with the 2004 National Institutes of Health/National Heart, Lung, and Blood Institute (NIH/NHLBI) guideline’s prevalence estimate during 2005–2008 and 2013–2016.

**Methods:**

This cross-sectional study analyzed the National Health and Nutrition Examination Survey data. High blood pressure included hypertension and elevated blood pressure (per the 2017 AAP guideline)/prehypertension (per the 2004 NIH/NHLBI guideline).

**Results:**

The analysis included 3,633 children in 2005–2008 and 3,471 children in 2013–2016. Per the 2004 NIH/NHLBI guideline, 3.1% (95% confidence interval [CI], 2.3%–4.3%) had hypertension in 2005–2008 and 1.9% (95% CI, 1.4%–2.6%) had hypertension in 2013–2016. Per the 2017 AAP guideline, prevalence was 5.7% (95% CI, 4.6%–7.1%) in 2005–2008 and 3.5% (95% CI, 2.7%–4.5%) in 2013–2016. About 2.5% (95% CI, 2.0%–3.1%) children in 2005–2008 and 1.5% (95% CI, 0.9%–2.0%) children in 2013–2016 were reclassified as hypertensive. We observed a similar change in prevalence for high blood pressure after application of the new guideline. The prevalence of high blood pressure also declined from 2005–2008 to 2013–2016 per both guidelines.

**Conclusion:**

Although the new guideline would reclassify a small proportion of children as having hypertension or high blood pressure, the prevalence declined from 2005–2008 to 2013–2016.

**Summary**

**What is already known on this topic?**

Changing the systolic/diastolic blood pressure cutoffs to define high blood pressure and hypertension in the 2017 American Academy of Pediatrics guideline could reclassify blood pressure levels as high blood pressure or hypertension.

**What is added by this report?**

About 2.5% children in 2005–2008 and 1.5% in 2013–2016 were reclassified as hypertensive; a similar proportion would be reclassified as having high blood pressure. The overall prevalence of both blood pressure levels declined from 2005–2008 to 2013–2016.

**What are the implications for public health practice?**

Increasing awareness is important to minimize burden and future complications of childhood hypertension.

## Introduction

Hypertension is the most common risk factor for cardiovascular disease among adults (1,2). Although there is no known direct link from hypertension to cardiovascular disease among children or adolescents, hypertension is associated with atherosclerosis, left ventricular hypertrophy, and renal complications among children and adolescents (3,4). Furthermore, childhood hypertension is associated with higher risks of cardiovascular disease during adulthood (3–6). A diagnosis may depend on which blood pressure level is used to define hypertension (7). In 2017, the American Academy of Pediatrics (AAP) Clinical Practice Guideline recommended new blood pressure levels to classify hypertension among children and adolescents. The new guideline, derived from an analysis that excluded overweight or obese children, lowered blood pressure levels by 2 to 3 mm Hg for children aged 12 years or younger on the basis of percentiles for age, sex, and height (8). Because the 90th and 95th blood pressure percentiles for adolescents are so close to the hypertension cut points for adults, the 2017 AAP guideline recommended the adult cut point of 130/80 mm Hg to diagnose hypertension among adolescents aged 13 years or older (8); the same cut point for adults was recommended by the revised hypertension guideline in 2017 from the American College of Cardiology/American Heart Association (ACC/AHA) (9).

On the basis of the 2017 AAP guideline, a larger proportion of children and adolescents than previously could be classified as having hypertension (7,8). An analysis of data from the National Health and Nutrition Examination Survey (NHANES) from 2011 through 2014 estimated that about 14% of adults in the United States would be classified as having hypertension as a result of applying the new ACC/AHA guideline (10). Sharma et al examined the consequences of this new blood pressure definition on the prevalence of hypertension among children (11). They analyzed NHANES data from 1999 through 2014 for 15,567 children and adolescents aged 5 to 18 years and found an overall change in hypertension prevalence from 11.8% to 14.2% after application of the new guideline (11). Although the study provided some insight into how the change in thresholds might affect the prevalence of hypertension among children, the analysis had limitations. In an accompanying editorial, Daniels noted the cross-sectional study design and the potential exclusion of children younger than 5 years in the NHANES data set (7). Furthermore, the authors analyzed all 16 years of data as a single period, an approach that did not allow a description of changes and trends in prevalence over time. Ma et al (12) showed that the prevalence of elevated blood pressure among US children trended downward during the same study period (1999–2014) investigated by Sharma et al (11). Likewise, Dorans et al (13) found a downward trend in the prevalence of hypertension among US adults after application of the 2017 ACC/AHA guideline. It is crucial to estimate an up-to-date prevalence of hypertension on the basis of recent data per the latest guideline instead of averaging the prevalence over 16 years (11). 

The prevalence of hypertension or high blood pressure could vary according to age, sex, and other sociodemographic characteristics among children and adults (10,11,13). Investigating trends among children after application of the new 2017 AAP guideline according to these sociodemographic characteristics is important. Considering the negative consequences of childhood hypertension, Healthy People 2020 (HP 2020) established a target to reduce childhood hypertension from its baseline value of 3.5% (2005–2008) to 3.2% by the year 2020 among children aged 8 to 17 years (14). Although several estimates based on the 2004 National Institutes of Health/National Heart, Lung, and Blood Institute (NIH/NHLBI) guideline suggest that the prevalence of hypertension or high blood pressure among US children is decreasing (12), it may be helpful to obtain estimates based on the 2017 AAP guideline to determine how changes in recommendations affect the prevalence of hypertension and high blood pressure. We aimed to fill these gaps in knowledge by applying the 2004 NHI/NHLBI and 2017 AAP guidelines to NHANES data to describe the prevalence and trend of blood pressure levels among children aged 8 to 17 in the United States.

## Methods

We used NHANES data collected during 2 periods: 2005–2008 and 2013–2016. NHANES is a biennial cross-sectional survey conducted since 1999 (15). The survey aims to obtain a nationally representative sample of noninstitutionalized people in the United States. Survey details are available elsewhere (15–17). NHANES data sets are publicly available from the Centers for Disease Control and Prevention (CDC) websites (18). We merged the data files for demographics and blood pressure with unique identification numbers to obtain both demographic and blood pressure measurements variables. Parents provided written informed consent for all children younger than 18. Children aged 8 to 11 years also provided assent. Written informed consent was obtained from children aged 12 years or older. The survey protocols were approved by the National Center for Health Statistics Research Ethics Review Board (19).

### Blood pressure measurements

NHANES measures blood pressure among participants 8 years or older. We limited our analysis to children aged 8 to 17 to conform to CDC’s HP 2020 objectives (14). Furthermore, the 2004 NIH/NHLBI guideline specifies this upper age limit (5). To categorize blood pressure levels among children aged 8 to 12, we used age-, sex-, and height-specific blood pressure percentiles recommended by the 2004 NIH/NHLBI guideline (5) and the 2017 AAP guideline (8). For children aged 13 to 17 years, we used percentiles recommended by the 2004 NIH/NHLBI guideline (5) and ≥130/80 mm Hg recommended by the 2017 AAP guideline (8). 

The 2004 NIH/NHLBI guideline defined prehypertension as a blood pressure from the 90th percentile to <95th percentile or if blood pressures were >120/80 mm Hg up to the 95th percentile. This guideline categorized stage 1 hypertension as the 95th to 99th percentile plus 5 mm Hg for systolic blood pressure/diastolic blood pressure and stage 2 hypertension as the >99th percentile plus 5 mm Hg (5,8,11). 

According to the 2017 AAP guideline, for children younger than 13, the cutoffs for elevated blood pressure were the 90th to <95th percentile or 120/80 mm Hg to <95th percentile (whichever was lower) (8,11). In the same age group per the 2017 AAP guideline, the cutoff to define stage 1 hypertension was the 95th to <95th percentile plus 12 mm Hg or 130/80 to 139/89 mm Hg (whichever was lower); the cutoff to define stage 2 hypertension was the ≥95th percentile plus 12 mm Hg or ≥140/90 mm Hg (whichever was lower) (8,11).

For children aged 13 or older, systolic blood pressure/diastolic blood pressure cutoff for elevated blood pressure was 120–130/<80 mm Hg, whereas the systolic blood pressure/diastolic blood pressure cutoff was 130-139/80-89 mm Hg for stage 1 hypertension and ≥140/90 mm Hg for stage 2 hypertension in the 2017 AAP guideline (8,11). 

Hypertension included stage 1 and stage 2 hypertension, whereas high blood pressure included both hypertension and elevated blood pressure (per the 2017 AAP guideline)/prehypertension (per the 2004 NIH/NHLBI guideline) (5,8,11). A child with at least 1 blood pressure measurement was eligible for our analysis. The average of the first 3 measurements was used for children with 3 or more blood pressure measurements. If a child had fewer than 3 measurements, the average of available blood pressure measurements was used to categorize blood pressure levels (20). A trained physician measured blood pressure with a calibrated sphygmomanometer in a mobile examination center. Appropriate cuff sizes were used. Blood pressure was measured while the child was in a seated position after a rest of 5 minutes (21).

### Sociodemographic variables

We grouped age into 2 categories: children aged 8 to 12 years and children aged 13 to 17 years. Participants reported their sex (ie, boy or girl). We used the following racial/ethnic categories: Mexican American, other Hispanic, non-Hispanic white, non-Hispanic black, and other. Body mass index (BMI) was calculated as weight in kilograms divided by height in meters squared. We used CDC’s age- and sex-specific BMI categories (22). We categorized a participant as overweight/obese if BMI was in the 85th percentile or above.

### Statistical analysis

We first tabulated data on sociodemographic and weight-related characteristics (age, sex, race/ethnicity, BMI, and whether overweight/obese) of the unweighted number of study participants. After using the Shapiro–Wilk test and determining that the data were not normally distributed, we described continuous variables as median and interquartile range (IQR). We tabulated categorical variables as number and percentage. We then compared the prevalence of blood pressure according to each guideline among both boys and girls in both survey periods. We also calculated the proportion of participants who would require further evaluation because of higher blood pressure levels per the 2017 AAP guideline in 2013–2016. In addition, we compared the prevalence estimate calculated for each guideline during both survey periods according to demographic and health-related characteristics. For all prevalence estimates, we adjusted for the multistage cluster-sampling design of the survey. We calculated all prevalence estimates and differences with 95% confidence intervals (CIs). All analyses were conducted by using Stata version 14.0 (StataCorp LLC).

## Results

Our analysis included 7,104 children with blood pressure measurements data: 3,633 from NHANES 2005–2008 and 3,471 from NHANES 2013–2016 (Table 1). For data collected in the 2005–2008 survey period, the 2004 NIH/NHLBI guideline classified 116 children as having hypertension, and the 2017 AAP guideline classified 197 children as such. For data collected in the 2013–2016 survey period, the 2004 NIH/NHLBI guideline categorized 84 children as having hypertension, and the 2017 AAP guideline classified 135 children as such. The median (IQR) age of the participants was 13 (10–15) years in 2005–2008 and 12 (10–15) years in 2013–2016. The 2004 NIH/NHLBI and 2017 AAP guidelines found the median (IQR) age among children with hypertension in 2005–2008 to be 12 (10–15) and 12 (10–16) years, respectively, which were slightly older than the median age among children with hypertension in 2013–2016 in both guidelines groups, the 2004 NIH/NHLBI and the 2017 AAP hypertensive groups as 10 (9–13) and 11 (9–14) years, respectively. 

**Table 1:** Characteristics of Children Surveyed, National Health and Nutrition Examination Survey, 2005–2008 and 2013–2016^a^

Characteristic	2005–2008			2013–2016		
	Overall (n = 3,633)	With Hypertension		Overall (n = 3,471)	With Hypertension	
		2004 NIH/NHLBI^b^ (n = 84)	2017 AAP^c^ (n = 135)		2004 NIH/NHLBI^b^ (n = 84)	2017 AAP^c^ (n = 135)
**Age, median (IQR), y**	13 (10–15)	12 (10–15)	12 (10–16)	12 (10–15)	10 (9–13)	11 (9–14)
**Age group, no. (%), y**						
8–12	1,370 (37.7)	47 (40.5)	79 (40.1)	1,565 (45.1)	55 (65.5)	87 (64.4)
13–17	2,263 (62.3)	69 (59.5)	118 (59.9)	1,906 (54.9)	29 (34.5)	48 (35.6)
**Sex**						
Male	1,823 (50.2)	54 (46.5)	102 (51.8)	1,758 (50.7)	45 (53.6)	83 (61.5)
Female	1810 (49.8)	62 (53.5)	95 (48.2)	1,713 (49.3)	39 (46.4)	52 (38.5)
**Race/ethnicity, no. (%)**						
Mexican American	1,077 (29.6)	31 (26.7)	48 (24.4)	749 (21.6)	26 (31.0)	36 (26.7)
Other Hispanic	268 (7.4)	6 (5.2)	8 (4.1)	405 (11.7)	12 (14.3)	17 (12.6)
Non-Hispanic white	1,049 (28.9)	34 (29.3)	61 (31.0)	960 (27.7)	16 (19.0)	33 (24.4)
Non-Hispanic black	1,057 (29.1)	41 (35.3)	71 (36.0)	835 (24.1)	22 (26.2)	31 (23.0)
Other	182 (5.0)	4 (3.5)	9 (4.6)	522 (15.0)	8 (9.5)	18 (13.3)
**BMI, median (IQR), kg/m^2^**	20.8 (18.0–24.8)	23.3 (19.8–28.5)	22.8 (19.4–27.6)	20.6 (17.7–24.4)	23.4 (19.7–26.5)	22.8 (19.1–26.5)
**Overweight/obesity, no. (%)**						
No	2,131 (59.1)	42 (37.2)	84 (43.7)	1,997 (58.2)	24 (29.3)	44 (34.7)
Yes	1,472 (40.9)	71 (62.8)	108 (56.3)	1,434 (41.8)	58 (70.7)	83 (65.3)
**Blood pressure, median (IQR)**						
Systolic	107 (100–113)	128 (122–133)	123 (117–131)	105 (99–112)	124 (119–131)	121 (117–129)
Diastolic	58 (50–65)	62 (54–72)	66 (55–80)	57 (49–64)	57 (46–67)	59 (46–74)
**Hypertension category among all participants**						
Normal blood pressure	NA	3,150 (86.7)	3,079 (84.8)	NA	3,137 (90.4)	3,084 (88.8)
Prehypertension or elevated hypertension	NA	367 (10.1)	357 (9.8)	NA	250 (7.2)	252 (7.3)
Stage 1 hypertension	NA	110 (3.0)	184 (5.1)	NA	80 (2.3)	120 (3.5)
Stage 2 hypertension	NA	6 (0.2)	13 (0.4)	NA	4 (0.1)	15 (0.4)
High blood pressure	NA	483 (13.3)	554 (15.2)	NA	334 (9.6)	387 (11.2)
Hypertension (overall)^d^	NA	116 (3.2)	197 (5.5)	NA	84 (2.4)	135 (3.9)

Abbreviations: AAP, American Academy of Pediatrics; BMI, body mass index; IQR, interquartile range; NA, not applicable; NIH/NHLBI, National Institutes of Health/National Heart, Lung, and Blood Institute.

a Percentages are column percentages unless otherwise indicated.

b National High Blood Pressure Education Program Working Group on High Blood Pressure in Children and Adolescents (5).

c Flynn and Falkner (8).

d Category combines stage 1 and stage 2 hypertension.

Although the proportion of boys and girls was similar overall among participants in both survey periods, the proportion of boys was higher among children with hypertension in all groups with hypertension except hypertensive children per the 2004 NIH/NHLBI guideline in 2005–2008 (Table 1). In 2005–2008, the greatest proportion of respondents were Mexican American (29.6%; n = 1,077) and non-Hispanic black (29.1%; n = 1,057), followed by non-Hispanic white (28.9%; n = 1,049), and other Hispanic or other race (12.4%; n = 450). The overall median BMI was similar in both study periods, and the median BMI among children with hypertension per both guidelines in both periods was higher than the overall median BMI. The proportion of overweight or obese children was higher in all 4 groups with hypertension than it was overall. Children with hypertension per both guidelines had higher median systolic blood pressure and diastolic blood pressure than the overall respondents in both survey periods. According to both guidelines, the proportion of participants with high blood pressure and hypertension was higher in 2005–2008 than in 2013–2016 (Table 1). Furthermore, the 2017 AAP guideline classified more children as having high blood pressure in both the 2005–2008 (15.2%; n = 554) and 2013–2016 (11.2%; n = 387) survey periods than did the 2004 NIH/NHLBI guideline (13.3%, n = 483 in 2005–2008; 9.6%, n = 334 in 2013–2016). Similarly, the proportion of participants with hypertension was higher per the 2017 AAP guideline in both survey periods (Table 1).

The prevalence of high blood pressure and hypertension was slightly higher per the 2017 AAP guideline than per the 2004 NIH/NHLBI guideline in both survey periods among both sexes (Table 2). The prevalence of high blood pressure and hypertension declined among both sexes between the 2 periods. Per the 2004 NIH/NHLBI guideline, the overall prevalence of high blood pressure declined from 13.4% (95% CI, 11.3%–15.9%) in 2005–2008 to 9.5% (95% CI, 8.2%–10.9%) in 2013–2016. In contrast, this prevalence declined from 15.2% (95% CI, 12.8%–17.9%) to 10.6% (95% CI, 9.3%–12.0%) per the 2017 AAP guideline. Overall, the prevalence of high blood pressure per the 2017 AAP guideline was 1.7 (95% CI, 1.2–2.2) percentage points higher than the prevalence per the 2004 NIH/NHLBI guideline in 2005–2008 and 1.1 (95% CI, 0.6–1.6) percentage points higher in 2013–2016. Likewise, the overall prevalence of hypertension decreased from 3.1% (95% CI, 2.3%–4.3%) in 2005–2008 to 1.9% (95% CI, 1.4%–2.6%) in 2013–2016 per the 2004 NIH/NHLBI guideline; this prevalence changed from 5.7% (95% CI, 4.6%–7.1%) in 2005–2008 to 3.5% (95% CI, 2.7%–4.5%) in 2013–2016 per the 2017 AAP guideline. The 2017 AAP guideline classified a greater percentage of children as hypertensive than did the 2004 NIH/NHLBI guideline: 2.5 percentage points (95% CI, 2.0–3.1) more in 2005–2008 and 1.5 (95% CI, 0.9–2.0) percentage points more in 2013–2016. Changes in prevalence were similar among boys and girls.

**Table 2:** Prevalence of Blood Pressure Levels, Stratified by Sex, Guideline Definition, and Survey Periods, National Health and Nutrition Examination Survey, 2005–2008 and 2013–2016^a^

Characteristic	2005–2008			2013–2016		
	2004 NIH/NHLBI^b^	2017 AAP	Difference, Percentage Point (95% CI)	2004 NIH/NHLBI^c^	2017 AAP	Difference, Percentage Point (95% CI)
**Boys**						
Normal blood pressure	83.7 (80.4 to 86.6)	82.0 (78.7 to 84.9)	−1.7 (−2.3 to 1.0)	88.2 (85.8 to 90.3)	87.0 (84.5 to 89.1)	−1.3 (−2.0 to 0.5)
Prehypertension or elevated blood pressure^d^	13.4 (10.9 to 16.4)	12.0 (9.6 to 14.7)	−1.5 (−2.8 to −0.1)	9.6 (7.8 to 11.9)	8.7 (7.1 to 10.6)	−0.9 (−2.0 to 0.2)
Stage 1 hypertension	2.8 (2.0 to 3.9)	5.6 (4.2 to 7.4)	2.8 (1.6 to 3.9)	2.0 (1.2 to 3.1)	3.9 (2.8 to 5.3)	1.9 (0.1 to 2.7)
Stage 2 hypertension	0.1 (0 to 0.3)	0.4 (0.2 to 1.3)	0.4 (−0.1 to 0.8)	0.1 (0 to 0.4)	0.5 (0.3 to 0.8)	0.3 (0.1 to 0.5)
High blood pressure^e^	16.3 (13.4 to 19.6)	17.9 (15.1 to 21.3)	1.7 (1.0 to 2.3)	11.7 (9.7 to 14.2)	13.0 (10.9 to 15.5)	1.3 (0.5 to 2.0)
Hypertension (overall)^f^	2.9 (2.0 to 4.0)	6.0 (4.5 to 8.1)	3.2 (2.0 to 4.3)	2.1 (1.3 to 3.2)	4.3 (3.2 to 5.8)	2.0 (1.4 to 3.0)
**Girls**						
Normal blood pressure	89.5 (86.5 to 91.9)	87.8 (84.4 to 90.5)	−1.8 (−2.8 to −0.8)	92.9 (91.2 to 94.3)	91.9 (90.1 to 93.4)	−0.9 (−1.5 to 0.3)
Prehypertension or elevated blood pressure^d^	7.0 (5.4 to 9.1)	6.9 (5.0 to 9.5)	0.1 (−1.8 to 1.5)	5.3 (4.1 to 6.9)	5.5 (4.0 to 7.4)	0.1 (−0.8 to 0.1)
Stage 1 hypertension	3.3 (2.1 to 5.3)	5.1 (3.8 to 6.8)	1.8 (0.7 to 2.8)	1.7 (1.1 to 2.5)	2.4 (1.7 to 3.4)	0.7 (0.1 to 1.3)
Stage 2 hypertension	0.1 (0 to 0.4)	0.2 (0.1 to 0.9)	0.1 (−0.1 to 0.4)	0.1 (0 to 0.9)	0.2 (0.1 to 0.7)	0.1 (−0.1 to 0.2)
High blood pressure^e^	10.4 (8.1 to 13.5)	12.2 (9.5 to 15.6)	1.8 (0.8 to 2.8)	7.1 (5.7 to 8.9)	8.0 (6.6 to 9.8)	0.9 (0.3 to 1.5)
Hypertension (overall)^f^	3.4 (2.1 to 5.4)	5.3 (4.0 to 7.1)	1.9 (0.9 to 2.8)	1.8 (1.2 to 2.6)	2.6 (1.9 to 3.6)	0.8 (0.2 to 1.4)
**Both**						
Normal blood pressure	86.6 (84.1 to 88.7)	84.8 (82.1 to 87.2)	−1.7 (−2.2 to −1.2)	90.5 (89.0 to 91.8)	89.4 (88.0 to 90.7)	−1.1 (−1.6 to −0.6)
Prehypertension or elevated blood pressure^d^	10.3 (8.7 to 12.1)	9.5 (7.8 to 11.4)	−0.8 (−1.6 to −0.1)	7.5 (6.4 to 8.9)	7.1 (6.0 to 8.3)	−0.4 (−0.1 to 0.4)
Stage 1 hypertension	3.1 (2.3 to 4.1)	5.4 (4.3 to 6.7)	2.3 (1.6 to 2.9)	1.8 (1.3 to 2.5)	3.1 (2.4 to 4.0)	1.3 (0.8 to 1.8)
Stage 2 hypertension	0.1 (0 to 0.2)	0.3 (0.2 to 0.8)	0.2 (0 to 0.5)	0.1 (0 to 0.5)	0.3 (0.2 to 0.6)	0.2 (0.1 to 0.3)
High blood pressure^e^	13.4 (11.3 to 15.9)	15.2 (12.8 to 17.9)	1.7 (1.2 to 2.2)	9.5 (8.2 to 10.9)	10.6 (9.3 to 12.0)	1.1 (0.6 to 1.6)
Hypertension (overall)^f^	3.1 (2.3 to 4.3)	5.7 (4.6 to 7.1)	2.5 (2.0 to 3.1)	1.9 (1.4 to 2.6)	3.5 (2.7 to 4.5)	1.5 (0.9 to 2.0)

Abbreviations: AAP, American Academy of Pediatrics; CI, confidence interval; NIH/NHLBI, National Institutes of Health/National Heart, Lung, and Blood Institute.

a Values are column percentage (95% CI) unless otherwise indicated.

b National High Blood Pressure Education Program Working Group on High Blood Pressure in Children and Adolescents (5).

c Flynn and Falkner (8).

d Prehypertension defined by 2004 NIH/NHLBI and elevated blood pressure defined by 2017 AAP guidelines.

e High blood pressure combines prehypertension, elevated blood pressure, stage 1 hypertension, and stage 2 hypertension.

f Category combines stage 1 and stage 2 hypertension.

The prevalence of both high blood pressure and hypertension decreased from 2005–2008 to 2013–2016 for most characteristics per both guidelines (Table 3). By characteristic, overweight or obese children had the highest prevalence of high blood pressure in both survey periods per both guidelines. The prevalence of high blood pressure among overweight or obese children was 18.3% (95% CI, 16.2%–20.6%) in 2005–2008 and 14.4% (95% CI, 12.3%–16.7%) in 2013–2016 per the 2004 NIH/NHLBI guideline. Per the 2017 AAP guideline, this prevalence was 20.9% (95% CI, 18.2%–24.0%) in 2005–2008 and 16.5% (95% CI, 14.2%–19.2%) in 2013–2016. Of the racial groups, the prevalence of high blood pressure was highest among non-Hispanic black children per both guidelines in both survey periods. After reclassification from the 2004 NIH/NHLBI guideline to the 2017 AAP guideline, the prevalence of high blood pressure increased for all characteristics except the 13- to 17-year age group.

**Table 3:** Prevalence of High Blood Pressure and Hypertension Among Children According to Selected Characteristics In 2 Survey Periods, National Health and Nutrition Examination Survey, 2005–2008 and 2013–2016^a^

Characteristic	2005–2008			2013–2016		
	2004 NIH/NHLBI^b^	2017 AAP^c^	Difference, Percentage Point (95% CI)	2004 NIH/NHLBI^b^	2017 AAP^c^	Difference, Percentage Point (95% CI)
**High Blood Pressure^d^**						
**Age group, y **						
8–12	9.2 (6.8 to 12.4)	13.9 (10.8 to 17.8)	4.7 (3.5 to 5.9)	7.4 (6.0 to 9.1)	10.5 (8.9 to 12.4)	3.1 (2.0 to 4.2)
13–17	16.0 (13.3 to 19.2)	15.9 (13.2 to 19.1)	−0.1 (−0.5 to 0.3)	10.8 (9.1 to 12.7)	10.6 (9.0 to 12.5)	−0.1 (−0.6 to 0.3)
**Sex **						
Male	16.3 (13.4 to 19.6)	18.0 (15.1 to 21.3)	1.7 (1.0 to 2.3)	11.8 (9.7 to 14.2)	13.0 (10.9 to 15.5)	1.3 (0.5 to 2.0)
Female	10.5 (8.1 to 13.5)	12.2 (9.5 to 15.6)	1.8 (0.8 to 2.8)	7.1 (5.7 to 8.9)	8.1 (6.6 to 9.8)	0.9 (0.3 to 1.5)
**Race/ethnicity**						
Mexican American	11.4 (8.5 to 15.3)	14.1 (10.1 to 19.1)	2.6 (0.9 to 4.3)	11.1 (8.8 to 13.8)	11.8 (9.8 to 14.1)	0.7 (−0.1 to 1.5)
Other Hispanic	11.2 (6.1 to 19.8)	12.5 (7.2 to 20.8)	1.2 (0.2 to 2.3)	10.4 (6.7 to 15.8)	12.8 (8.8 to 18.3)	2.5 (1.0 to 3.9)
Non-Hispanic white	13.6 (10.7 to 17.1)	15.1 (11.9 to 19.0)	1.5 (0.6 to 2.4)	8.3 (6.5 to 10.6)	9.2 (7.3 to 11.4)	0.9 (0 to 1.7)
Non-Hispanic black	15.9 (13.3 to 18.9)	18.1 (15.1 to 21.6)	2.2 (1.2 to 3.3)	12.2 (10.3 to 14.4)	13.8 (11.7 to 16.2)	1.6 (0.9 to 2.4)
Other	12.4 (7.0 to 21.0)	13.8 (8.1 to 22.5)	1.4 (−1.0 to 3.8)	9.2 (6.4 to 13.2)	10.5 (7.6 to 14.4)	1.3 (0.5 to 2.2)
**Overweight/obesity**						
No	10.1 (7.5 to 13.4)	11.3 (8.4 to 15.1)	1.2 (0.6 to 1.9)	5.7 (4.5 to 7.2)	6.1 (5.0 to 7.5)	0.4 (−0.1 to 1.0)
Yes	18.3 (16.2 to 20.6)	20.9 (18.2 to 24.0)	2.6 (1.4 to 3.9)	14.4 (12.3 to 16.7)	16.5 (14.2 to 19.2)	2.2 (1.3 to 3.1)
**Hypertension^e^**						
**Age group, y**						
8–12	3.6 (2.4 to 5.4)	6.0 (4.5 to 7.9)	2.3 (1.3 to 3.3)	3.0 (2.1 to 4.3)	5.5 (4.1 to 7.3)	2.5 (1.5 to 3.4)
13–17	2.8 (1.8 to 4.4)	5.5 (4.1 to 7.3)	2.7 (1.7 to 3.7)	1.3 (0.8 to 2.0)	2.2 (1.5 to 3.2)	0.9 (0.3 to 1.6)
**Sex**						
Boys	2.9 (2.1 to 4.0)	6.0 (4.5 to 8.1)	3.2 (2.0 to 4.3)	2.1 (1.4 to 3.2)	4.3 (3.2 to 5.8)	2.3 (1.4 to 3.0)
Girls	3.4 (2.2 to 5.4)	5.3 (4.0 to 7.1)	1.9 (1.0 to 2.9)	1.8 (1.3 to 2.6)	2.6 (1.9 to 3.6)	0.8 (0.2 to 1.4)
**Race**						
Mexican American	2.6 (1.5 to 4.5)	4.4 (2.9 to 6.8)	1.8 (0.8 to 2.8)	3.2 (2.3 to 4.5)	4.4 (2.9 to 6.5)	1.1 (0.1 to 2.2)
Other Hispanic	3.3 (1.1 to 9.4)	4.3 (1.7 to 10.3)	1.0 (−0.6 to 2.6)	3.2 (2.0 to 5.0)	4.7 (2.3 to 9.5)	1.6 (−0.7 to 3.9)
Non-Hispanic white	3.3 (2.1 to 5.1)	6.0 (4.3 to 8.3)	2.7 (1.8 to 3.7)	1.4 (0.8 to 2.5)	3.0 (2.1 to 4.5)	1.6 (0.8 to 2.5)
Non-Hispanic black	3.9 (2.8 to 5.3)	6.9 (5.0 to 9.5)	3.0 (1.5 to 4.6)	2.5 (1.3 to 4.7)	3.6 (2.1 to 6.1)	1.2 (−0.1 to 2.4)
Other	1.5 (0.4 to 5.9)	3.8 (1.8 to 8.0)	2.3 (0.5 to 4.1)	1.3 (0.6 to 2.7)	3.4 (2.0 to 5.9)	2.1 (0.6 to 3.7)
**Overweight/obesity**						
No	2.5 (1.5 to 4.0)	4.7 (3.1 to 7.0)	2.2 (1.2 to 3.2)	1.2 (0.8 to 1.7)	2.2 (1.5 to 3.1)	1.0 (0.4 to 1.5)
Yes	4.2 (3.1 to 5.7)	7.3 (5.7 to 9.3)	3.1 (1.8 to 4.5)	3.1 (2.1 to 4.5)	5.0 (3.5 to 7.1)	1.9 (0.8 to 3.1)

Abbreviations: AAP, American Academy of Pediatrics; CI, confidence interval; NIH/NHLBI, National Institutes of Health/National Heart, Lung, and Blood Institute.

a Values are percentage (95% CI) unless otherwise indicated.

b National High Blood Pressure Education Program Working Group on High Blood Pressure in Children and Adolescents (5).

c Flynn and Falkner (8).

d High blood pressure combines prehypertension, elevated blood pressure, stage 1 hypertension, and stage 2 hypertension.

e Hypertension combines stage 1 and stage 2 hypertension.

The prevalence of hypertension was similar across most characteristics per the 2004 NIH/NHLBI guideline in 2005–2008 (Table 3). The prevalence of hypertension was lower in 2013–2016 than in 2005–2008 for all characteristics except Mexican American race per both guidelines. By characteristic, similar to the prevalence of high blood pressure, the prevalence of hypertension was highest among overweight or obese children in 2005–2008: 4.2% (95% CI, 3.1%–5.7%) per the 2004 NIH/NHLBI guideline and 7.3% (95% CI, 5.7%–9.3%) per the 2017 AAP guideline. In 2013–2016, by characteristic, the prevalence of hypertension was highest among Mexican American children (3.2% [95% CI, 2.3%–4.5%]) and other Hispanic children (3.2% [95% CI, 2.0%–5.0%]) per the 2004 NIH/NHLBI guideline. The prevalence of hypertension was highest among children aged 8 to 12 years per the 2017 AAP guideline in 2013–2016 (5.5% [95% CI, 4.1%–7.3%]). After application of the new guideline, the estimated change in prevalence of hypertension ranged from 1.0% to 3.2% in 2005–2008 and 0.8% to 2.5% in 2013–2016. 

Per the 2017 AAP guideline, 18.9% (95% CI, 17.3%–20.7%) of children could require further evaluation (Table 4). The proportion of children who would require further evaluation because of the new 2017 AAP guideline was higher among boys than girls across all age groups.

**Table 4:** Percentage (95% Confidence Interval) of Children Requiring Further Evaluation of Blood Pressure Levels According to the 2017 AAP Guideline^a^, National Health and Nutrition Examination Survey, 2013–2016

Age, y	Boys	Girls	Both
8	19.4 (14.8–24.9)	18.6 (12.9–25.9)	19.0 (15.1–23.6)
9	27.4 (21.8–33.7)	25.2 (20.4–30.6)	26.2 (22.3–30.6)
10	30.5 (24.9–36.6)	26.9 (21.1–33.5)	28.8 (24.5–33.5)
11	28.2 (22.6–34.4)	25.5 (18.8–33.6)	26.8 (22.3–32.0)
12	23.3 (18.1–29.5)	21.0 (15.8–27.4)	22.3 (18.2–26.9)
≥13	19.0 (16.6–21.7)	8.3 (6.5–10.5)	13.7 (12.0–15.6)
Overall	22.3 (20.2–24.5)	15.5 (13.5–17.7)	18.9 (17.3–20.7)

Abbreviation: AAP, American Academy of Pediatrics.

a Flynn and Falkner (8).

## Discussion

We investigated the prevalence and trends of hypertension and high blood pressure among the US pediatric population in 2 survey periods with 2 blood pressure guidelines. After comparing the prevalence of the 2017 AAP guideline with the prevalence of 2004 NIH/NHLBI guideline, we found a small proportion of children would be reclassified as having high blood pressure or hypertension among both sexes and across a range of characteristics. About 1 in 5 children would also require additional evaluation because of high blood pressure during the NHANES examination. Furthermore, the prevalence of high blood pressure was lower in the second survey period than in the first survey period.

Despite a slightly different age group and different survey periods, the prevalence of hypertension and high blood pressure in our study is similar to the prevalence observed by earlier studies that investigated the prevalence of these conditions among children and adolescents in the United States (11,12,20,23). Those studies also found a modest increase in the prevalence of raised blood pressure levels after application of the new 2017 AAP guideline (11,23). Additionally, 1 study observed a decline in prevalence of high blood pressure and hypertension among children similar to the decline we observed (23). Antihypertensive medication use and improvement of dietary habits could have contributed to the decline in prevalence of childhood hypertension (11,23).

According to both guidelines, all children with higher-than-normal blood pressure levels would require further evaluation as well as early intervention (5,8). Hypertension during childhood could lead to a higher risk of hypertension and other cardiovascular diseases during adulthood (3,6). Furthermore, childhood hypertension is often unrecognized because symptoms of hypertension are relatively uncommon among children, compared with adults, and complications usually occur several years after onset (5,8). We recommend further evaluation of children with high blood pressure and children who could be recommended for further evaluation by the 2017 AAP guideline (8).

Although the prevalence of hypertension per the 2004 NIH/NHLBI guideline (1.9%) was lower than the HP 2020 target of 3.2% in 2013–2016, the 2017 AAP prevalence estimate was higher (3.5%) (14). Populations with a higher prevalence of childhood hypertension need to be reached with awareness, prevention, and control programs. Previous studies that investigated awareness and medication use found that a small proportion of children with hypertension received an early diagnosis or treatment (24,25). It is important to increase screening among children to avoid the negative consequences of raised blood pressure. Furthermore, the 2017 AAP guideline recommends annual screening of all children aged 3 years or older for blood pressure levels (8). Implementing such a recommendation is essential to achieve the HP 2020 target under the new guideline (14). Childhood hypertension prevention and control programs should focus on raising awareness among children, adolescents, caregivers, and physicians to monitor blood pressure levels.

Our study has several notable strengths. First, our prevalence estimates and trends were derived from 4 large nationally representative samples. NHANES used a standardized validated method to report blood pressure levels. We also reported prevalence estimates according to several characteristics.

Our study also has several limitations. The true population prevalence of hypertension could be lower than reported in our study because blood pressure was measured on a single day rather than during multiple visits. Some misclassification could also have resulted from “white-coat” hypertension (26). The small sample size for characteristics such as age, sex, and racial groups may have also caused additional error in estimating prevalence for subgroups.

This study examined the prevalence of hypertension and high blood pressure among children and adolescents. Although the prevalence was lower in 2013–2016 than in 2005–2008, and the new guideline could reclassify a small proportion of the pediatric population as having hypertension, a significant proportion of children would require further evaluation. Considering the negative consequences of hypertension, it is important to raise awareness among physicians and caregivers about childhood hypertension.
